# In the Laboratory and during Free-Flight: Old Honey Bees Reveal Learning and Extinction Deficits that Mirror Mammalian Functional Decline

**DOI:** 10.1371/journal.pone.0013504

**Published:** 2010-10-19

**Authors:** Daniel Münch, Nicholas Baker, Claus D. Kreibich, Anders T. Bråten, Gro V. Amdam

**Affiliations:** 1 Department of Chemistry, Biotechnology and Food Science, Norwegian University of Life Sciences, Aas, Norway; 2 School of Life Sciences, Arizona State University, Tempe, Arizona, United States of America; University of Alabama, United States of America

## Abstract

Loss of brain function is one of the most negative and feared aspects of aging. Studies of invertebrates have taught us much about the physiology of aging and how this progression may be slowed. Yet, how aging affects complex brain functions, e.g., the ability to acquire new memory when previous experience is no longer valid, is an almost exclusive question of studies in humans and mammalian models. In these systems, age related cognitive disorders are assessed through composite paradigms that test different performance tasks in the same individual. Such studies could demonstrate that afflicted individuals show the loss of several and often-diverse memory faculties, and that performance usually varies more between aged individuals, as compared to conspecifics from younger groups. No comparable composite surveying approaches are established yet for invertebrate models in aging research. Here we test whether an insect can share patterns of decline similar to those that are commonly observed during mammalian brain aging. Using honey bees, we combine restrained learning with free-flight assays. We demonstrate that reduced olfactory learning performance correlates with a reduced ability to extinguish the spatial memory of an abandoned nest location (spatial memory extinction). Adding to this, we show that learning performance is more variable in old honey bees. Taken together, our findings point to generic features of brain aging and provide the prerequisites to model individual aspects of learning dysfunction with insect models.

## Introduction

In populations with increased life expectancies, such as modern human societies and captive mammals, cognitive dysfunction is a prominent feature of aged cohorts. Surveys of brain aging in mammals commonly reveal two observations [1]. First, aging can affect several cognitive skills concomitantly. Second, the progression of cognitive aging can be highly variable, resulting in an increased heterogeneity of cognitive abilities among aged individuals [2], [3]. It is still not well understood how different features of aging are associated with each other [4], and the mechanisms that cause increased performance heterogeneity in aged groups are debated. This heterogeneity has often been attributed to the onset of multiple yet different pathologies [5], as well as ceasing mental- and physical activities. However, the orchestrated emergence of pathologies and reduced activity in the elderly could potentially also drive a more uniform pattern of decline [1]. Recent theories on aging, therefore, have invoked stochastic mechanisms to explain heterogeneity [6].

The opportunity to model the complex characteristics of mammalian brain aging has received little attention by research on invertebrate model organisms, which otherwise have greatly expanded our knowledge of how life span is influenced by molecular signaling networks [7]–[9] and socio-environmental factors [10], [11]. However, behavioral aging has been shown for the fruit fly, *Drosophila melanogaster*, and the honey bee, *Apis mellifera* [12]–[15], and paradigms comparable to those used to study mammalian brain aging can be applied to insects [16].

Among insect laboratory organisms, the honey bee represents one of the best developed restraint model that allows quantification of individual performance in memory acquisition tasks. Also, because honey bees express a rich and well-characterized repertoire of complex behaviors, such as extinction learning, stimulus categorization, rule learning, and advanced navigation [17]–[19], they provide an excellent opportunity for examining generic features of brain aging.

In classical conditioning paradigms, restrained honey bees readily learn to associate a neutral odor or shape (conditioned stimulus, CS) with a sucrose reward (unconditioned stimulus, US) [20], and can form several memory types including short-term, mid-term and long-term memory (LTM, early and late) [21]. This learned behavioral response, furthermore, is not fixed but can be actively extinguished. Memory extinction is appealing to experimentalists because it enables animals to respond appropriately under changing conditions [22], [23]. In extinction learning paradigms, individuals are tested for their ability to extinguish an acquired conditioned response, when the previously learned stimulus is no longer rewarded with the US [24]. Honey bees do not simply delete the acquired CS-US association during extinction (“forgetting”) [25], [26]. Rather, for honey bees as for mammals, extinction represents the consolidation and complex interaction of two opposing memories, the newly formed CS-noUS and the previously learned CS-US association [27].

In mammals, the capacity to extinguish memory declines during aging, which can cause several forms of distress [22]. For these models, the effect of aging on extinction learning is primarily tested in spatial learning paradigms that allow animals to move freely [23]. Free-flight systems for testing extinction learning are not equally established for honey bees, but relevant tools have been developed to test flight behavior towards the nest (“homing”) [28] or artificial foraging sites [29]. Generating artificial swarms and subsequent displacement of entire honey bee colonies, moreover, indicate that bees may learn to extinguish memory of a previous nest location [30]. We reasoned that a similar approach would allow us to assess age related differences in extinction of spatial memory simultaneously in a large number of bees. By quantifying the abilities of honey bees in combined laboratory and free-flight experiments, furthermore, we could test if an insect can show aging phenotypes that share features with mammalian brain aging.

Honey bees are characterized by a temporal polyethism among sister workers that pass through a sequence of well-defined social tasks, i.e. nest and foraging tasks. In particular, studies on behavioral aging repeatedly detected reduced olfactory memory acquisition in bees after 15 days of foraging, compared to bees that were performing nest tasks [14], [15]. Therefore, in our experiment we used bees that engaged in foraging, a task that is accompanied by a fast progression of symptoms that are characteristic of aging [10], [31]–[33]. In foragers, we quantified behavioral performance values while bees were restrained in the laboratory and flying under natural conditions, thus combining surveys of simple associative learning with a more complex spatial extinction task.

Our results suggest that older foragers, on average, are less capable of expressing new memory that contradicts previously learned memory. This finding indicates that complex extinction abilities can be affected during aging in invertebrates. Also, as shown previously for mammals, old honey bees were characterized by significantly higher inter-individual variance (heterogeneity) of memory acquisition performance compared to controls. This study shows how elementary principles of mammalian behavioral aging can be modeled in insects.

## Results

### Experiment 1: In-lab testing of olfactory learning, long-term memory retention, and extinction learning of old forager bees and mature controls

To assess age related decline of olfactory memory performance, we first contrasted two experimental groups with different foraging ages: mature *controls* were collected 5–10 days after the onset of foraging, *old* forager bees were allowed to forage for more than 15 days. Foraging age was tracked by individual paint-marks, as described previously [14]. Old foragers and mature controls were collected together over four replicate days. For the first two replicate days both groups were collected together from two colonies, while another two colony sources were used for the last two replicate days.

**Figure 1:**
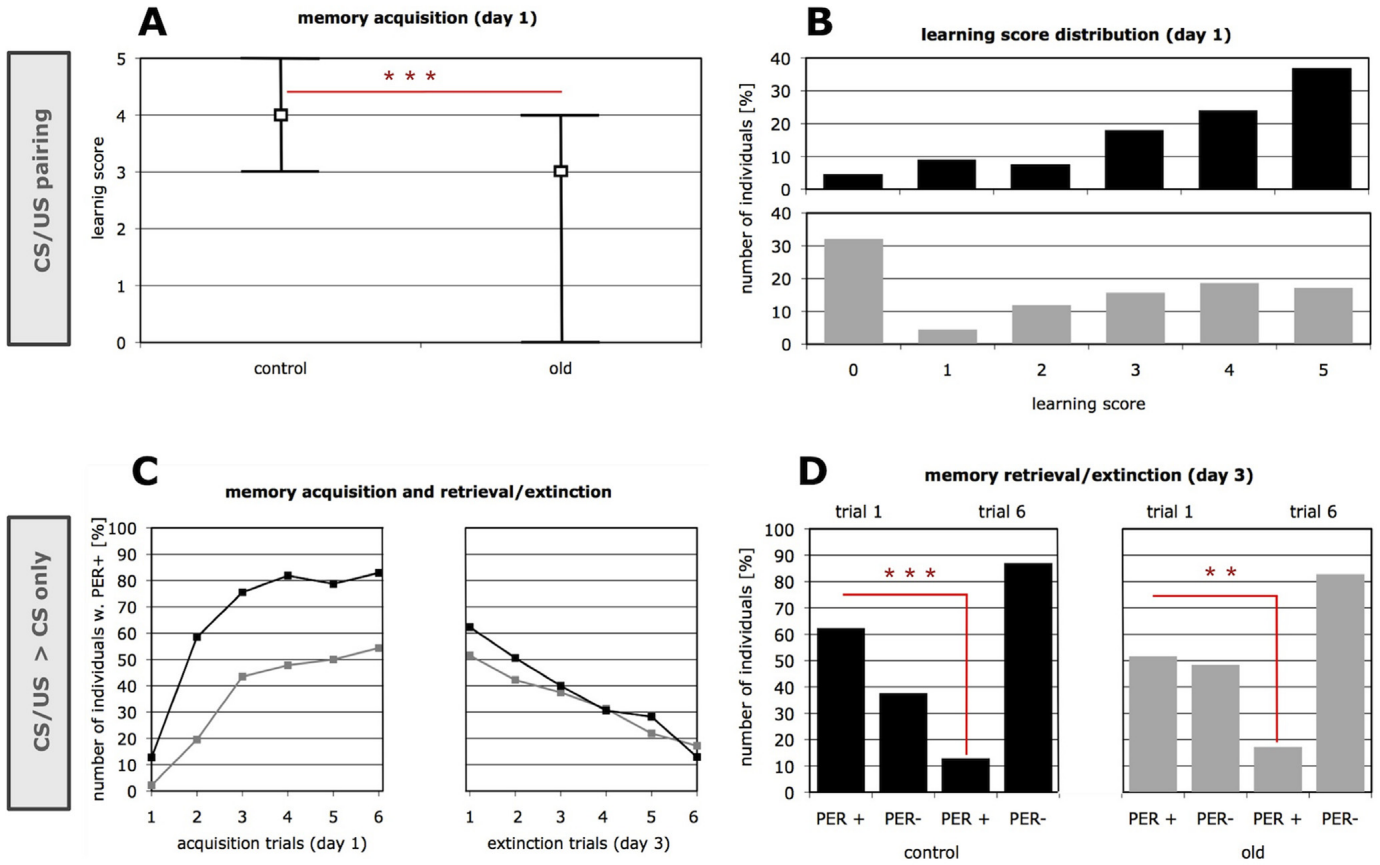
Acquisition, memory retention and extinction in old forager bees as compared to mature controls. The foraging durations in the two test groups were either less than 10 days (control) or longer than 15 days (old). (**A**) The learning performance in the old group was significantly reduced, as compared to the mature controls. Higher LS (up to LS = 5) indicate good learning performance, while lower LS indicate positive responses to the conditioned stimulus (CS, carnation oil) only in few or none (LS = 0) of the CS-US pairings. The graph shows medians and interquartile ranges with n = 133/134 for control and old, respectively. (**B**) Reduced learning performance in the old group is contrasted by increased performance heterogeneity (F = 1.65, p = 0.002, df_1_ = 132, df_2_ = 133, F-test; compare also interquartile ranges in A). Histograms of individual learning scores with n = 133/134 for control and old, respectively). (**C**) Acquisition, memory retention and extinction. To test acquisition a subset of bees was subjected to 6 CS-US pairings on day 1 (left). On day 3 bees were presented 6 times with the CS alone for testing memory retention (1^st^ trial) and extinction (response decay in the 6^th^ as compared to 1^st^ trial). The *y*-axis displays the percentage of individuals that responded to the CS by extending the proboscis (PER+). Day 1 with n = 94/92, day 3 with n = 85/64 for control and old, respectively. Differences in individual numbers between day 1 and 3 are mainly caused by mortality, specifically affecting the group of old foragers. (**D**) No significant difference in memory retention was detected when comparing the response of the two age groups to the first CS only presentation (n = 85/64 for control and old, respectively). After six extinction trials, PER- individuals do not respond to the learned CS-US association, and thereby show extinction. While response decline after extinction trials was less significant in the old group, a direct comparison of both groups does not reveal a significant age affect for extinction of olfactory memory (for details see results section). Asterisks in A, B, D denote significance (A, Chi-square; B, Mann-Whitney U; D, McNemar χ^2^).

For appetitive olfactory acquisition, bees were trained using six odor-sucrose (CS-US) pairings to assess olfactory learning performance. We found that foraging age explained the variation in memory acquisition (Fig. 1A, B). As previously established [14], the median learning score (LS) of bees that foraged for >15 days (old) was significantly lower than for the control (Mann-Whitney U-test Z = -5.24, p<0.001, n = 133/134, df = 1 for control/old, respectively). Also, fewer old forager bees expressed the learned response to the CS-US association (Chi-square test: χ^2^ = 33.88, p<0.001, n = 133/134, df = 1), where those showing a conditioned behavior showed at least one response (LS≥1) by extending the proboscis (PER+, proboscis extension response), and those not showing the conditioned behavior never responded to the CS alone (LS = 0). Furthermore, an F-test of variance established that old bees were significantly more heterogeneous in their performance than the control (F = 1.65, p = 0.002, df_1_ = 132, df_2_ = 133). This increased heterogeneity due to longer foraging is illustrated by larger interquartile ranges for the group of old foragers in Fig. 1A, and by histograms for learning performance values (LS) in Fig. 1B.

Collection day and source colony (i.e. replicate effects), in contrast, did not influence acquisition performance (Kruskal-Wallis ANOVA for learning performance: factor = day; H = 4.37, p = 0.22, n = 94/78/54/41; factor = colony; H = 6.30, p = 0.10, n = 86/86/33/62).

A subset of bees was also tested for memory retention and extinction on day 3 (Fig. 1C, right), i.e. two days after bees were trained with the 6 CS-US pairings on day 1 (Fig. 1C, left). Bees were presented once with the unrewarded odor (CS) to test for retention of LTM. Thereafter, five more unrewarded CS presentations were applied to extinguish the CS-US association that was learned on day one (Fig. 1C).

Again, the median learning score for acquisition on day 1 was significantly lower in old foragers (Mann-Whitney U-test Z = −5.46, p<0.001, n = 82/90, df = 1,when bees with spontaneous response to the CS in trial 1 were excluded as before; Z = −6.05, p<0.001, n = 94/92, df = 1, when LS was calculated for all bees that were to be tested on day3). We could not detect a significant difference in memory retention between old foragers and mature controls (Fig. 1C, D) when comparing the response to the first unrewarded CS presentation on day 3. In effect, the frequency of bees that showed memory retention with PER+ as compared to bees with no response to the CS (PER−) was similar in both groups (χ^2^ = 1.74, p = 0.19, n = 85/64 for control/old, respectively; Fig. 1D).

Presenting six times the CS only (learned odor without sucrose reward) to induce extinction, led to a significant response decline in both age groups. We observed a smaller response decline for old foragers, a possible indication for poorer extinction in this group (Fig. 1C, right). This is conveyed by different significance levels, when comparing the number of individuals with PER+ to the CS only in trial 1 and trial 6 (McNemar, χ^2^ = 26.27, p<0.001, n = 53, df = 1 for control; χ^2^ = 10.02, p = 0.002, df = 1, n = 33 for old, Fig. 1D). However, a direct comparison of the response decline during extinction does not reveal significant differences between the groups (χ^2^ = 0.89, p = 0.32, n = 53/33 for control/old, respectively). Thus, unlike acquisition performance and performance heterogeneity on day 1, our experiments on restrained honey bees cannot verify that aging affects memory consolidation and extinction.

One limitation of testing potentially short-lived individuals on successive days, however, is the uneven removal of animals from the experimental groups, which were of different foraging age. In fact, mortality was considerably higher in the old group (24.5% in old vs. 4.8% in control). Further, a recent study showed that individuals with poorer learning performance - typically enriched in groups of old foragers (Fig. 1B) - survive for a shorter time period, when challenged by stressors in a laboratory assay [34]. Therefore, significant age differences, as shown for memory extinction in other species, might have been concealed by an enrichment of less frail and better performing animals, that specifically affects the pool of old foragers in our restrained tests.

### Experiment 2: “Homing” performance of aged bees during free-flight correlates with olfactory learning performance in the laboratory

**Figure 2:**
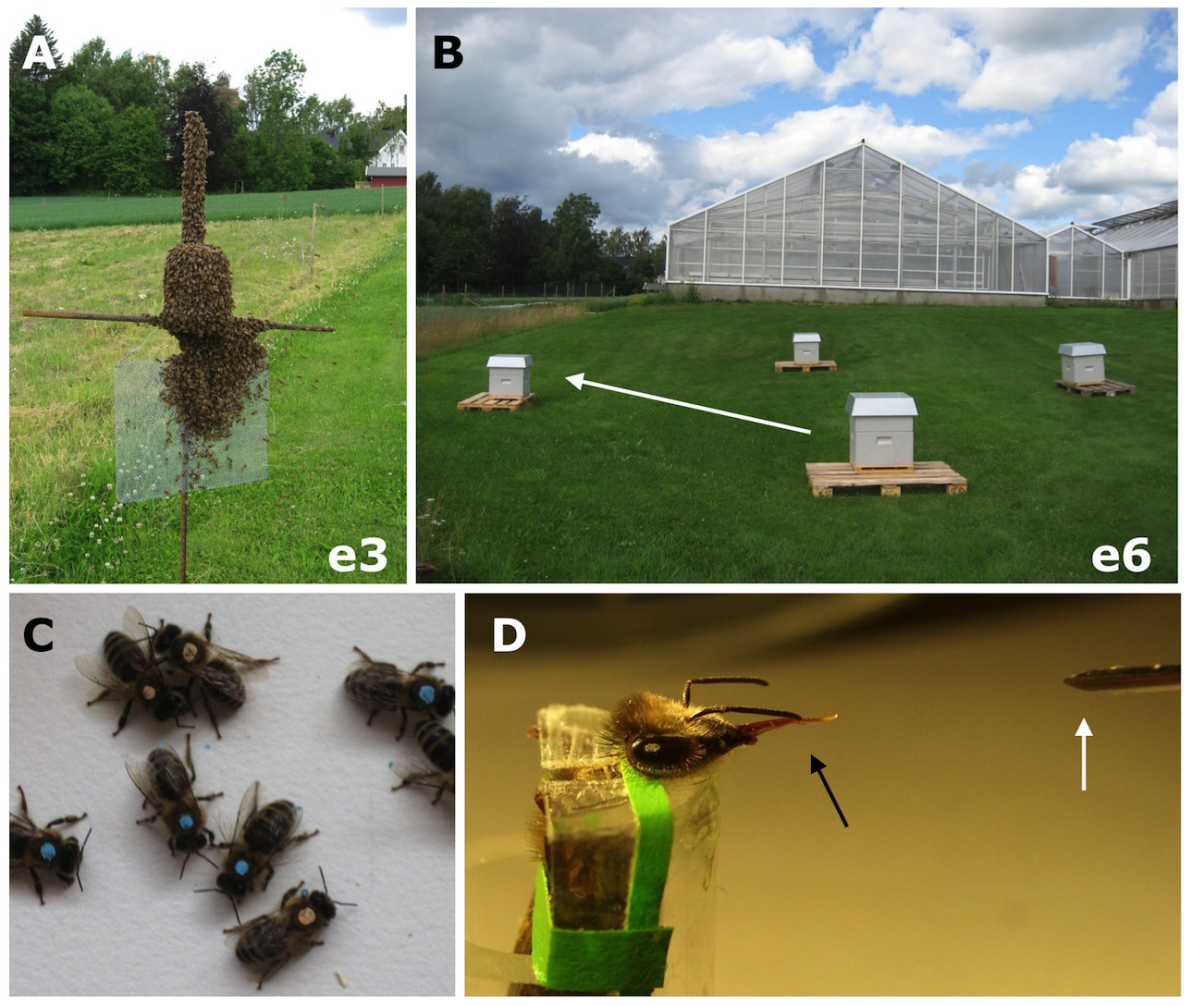
Relating individual performance in unlike memory tasks under free-flight and restrained conditions. (**A**) Artificial swarm with foragers attracted to a queen. The queen is caged and attached to an iron cross. Swarms, as shown here, were produced prior to moving entire colonies to a new location within an arena of four similar hive boxes (**B**). Spatial extinction was tested in three separate arenas with four hive boxes. One arena is exemplified here. Colony translocations (white arrow) were used to test the ability of aged bees to extinguish the spatial memory of a previous hive location. (**C**) Paint marks were applied to track individual foraging age and colony source. (**D**) In the laboratory, differences in acquisition of olfactory memory were quantified by monitoring the proboscis extension response (PER, black arrow) to the CS (odor, white arrow) during a sequence of 6 CS-US pairings. (e3 and e6 refer to events illustrated in Fig. 3).

In an experiment that overcomes this shortcoming, we tested whether inter-individual variation of performance decline was linked to variation in complex spatial extinction during free-flight (for illustration see Fig. 2D respectively 2B).

**Figure 3:**
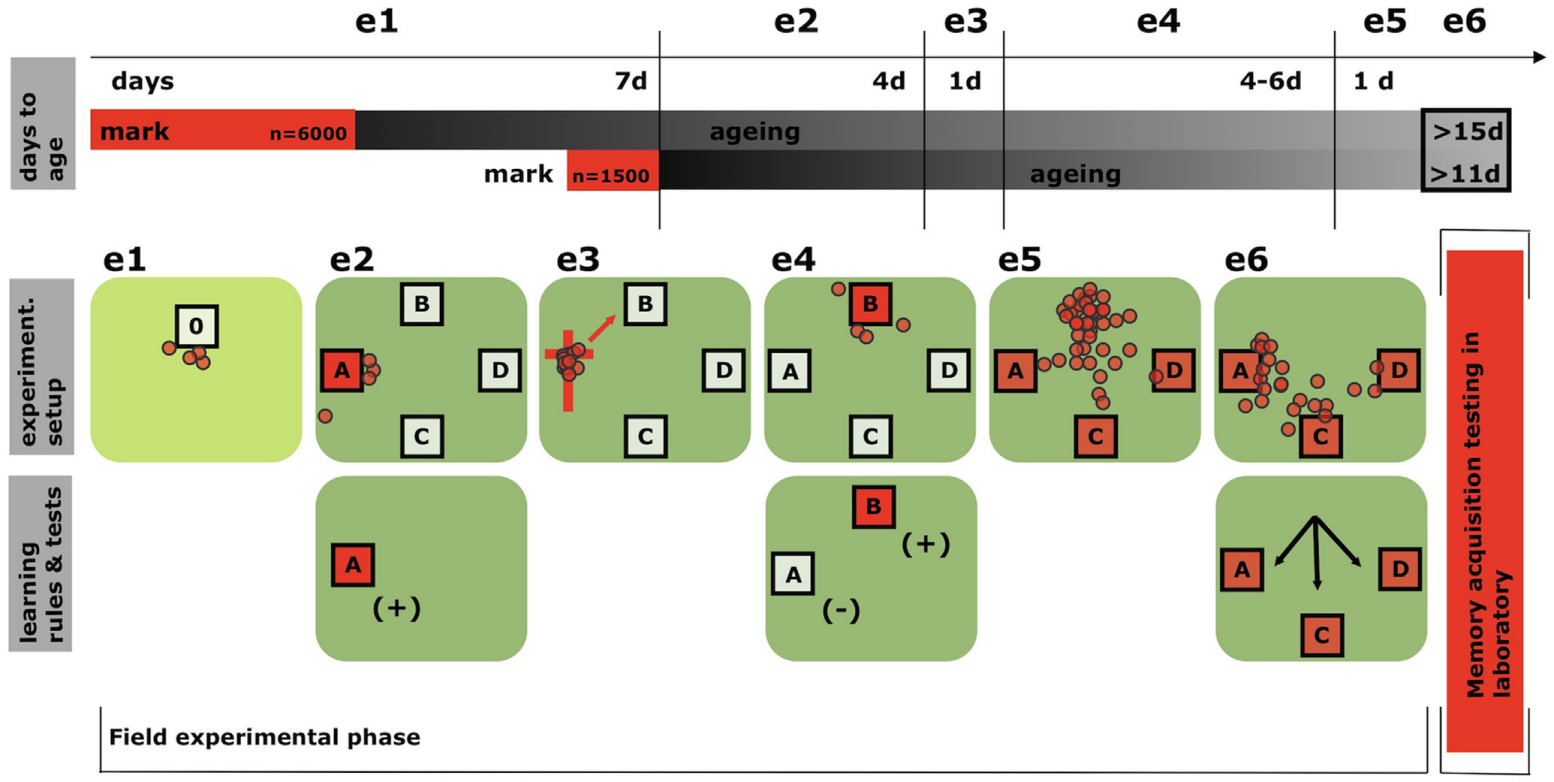
Experimental design used for inducing spatial memory extinction of a nest site and relating this extinction performance with olfactory learning decline. e1–e6 denote the separate events that constituted the spatial memory experiment. The upper row “days to age” depicts the overall timeline of the experiment. To control for foraging duration, foragers of random age were marked in two rounds. The initial day of olfactory learning performance scoring, these foragers constituted a heterogeneous age cohort with fully mature to old individuals that had been foraging for more than 15 respectively 11 days. The middle (“experiment. setup”) and lower rows (“learning rules and tests”) show the sequence of events that were used to induce spatial memories of the different nest sites. Bees returning from foraging flights were marked (event e1). Subsequently, they were moved to a distant test arena with four hive boxes (e2). Except for A, all other hive boxes (‘dummies’) were empty. After foraging, bees were given 4 days for learning to orient towards A. Thereafter, an artificial swarm was produced (e3, compare Fig. 2A) and was moved to location B (e4). Colonies were then given another 4–6 days to learn the spatial setting of the new home site B, while learning to extinguish the memory of the previous home site A, which remained closed (unrewarded ‘dummy’ location). Lastly, the entire worker population of the colony was dumped to the ground (e5). The hive box at B was removed, forcing foragers to orient towards the previous location A or alternative locations C, D (e6). At this time all locations A, C, D were similarly equipped with unrelated queens, young workers (<48 h old) and combs to resemble functioning hives. The next day, marked bees were collected, individual orientation preference was logged, and specimens were subjected to olfactory memory acquisition tests in the laboratory.

In contrast to experiment 1, we here generated a single, mixed-age population of well mature to old foragers, wherein considerable heterogeneity of performance was expected (see Fig. 1B). This was achieved by marking bees of random foraging age in two rounds, so that on testing days the bees of this mixed-age population had been foraging for 15 days or more (first marking), and 11 days or more (second marking, see Fig. 3, “days to age”).

In three replicates of the experiment, a colony comprised of paint-marked bees was moved from a distant apiary (Fig. 3. event e1) to an arena with nest boxes (Fig. 2b). The four boxes were oriented perpendicularly, facing each other in a north-south, east-west fashion (Fig. 3.e2). The focal colony of each replicate was located at box A, while the other boxes (B, C, D) remained empty and closed. At A, the bees were given several days to learn the spatial setting of their home range (timeline in Fig. 3). Next, they were shaken into a swarm (Fig. 3.e3 and Fig. 2A) and hived in box B, while box A was closed (Fig. 3.e4). The colony was now trained to the novel location B (“rewarded”, colony home) while the previous location A remained closed (no colony). A group of foragers from the original distant apiary was added to the swarm before it was hived at the novel location B. These bees were naïve to the previous nest location A (they had never experienced it) and served as a control. Lastly, open trap boxes with queens, brood and young workers (<24 h old) were set up at locations A, C and D. All bees in box B were brushed to the ground and box B was removed (Fig. 3.e5). Individual foragers were now forced to choose between the extinguished location A and the alternative, but identical boxes C and D. Trapped bees of the marked, mixed-age population were collected from A, C, and D the next morning (Fig. 3.e6). The collected individuals were subsequently scored for behavioral performance in the laboratory (for an illustration of classical conditioning using the PER, see Fig. 2D).

We found that across the three replicates, a greater number of marked age tracked foragers had entered the box at A (n = 55) compared to boxes at locations C (n = 26) and D (n = 3). This pattern of preferential orientation toward A, the previous nest location, was consistent over the replicates and was significantly different from an H_0_ expectation of equal orientation toward A vs. C and D (replicate 1, χ^2^ = 16.92, p<0.001, df = 1; replicate 2, χ^2^ = 9.07, p = 0.011, df = 1; replicate 3, χ^2^ = 12.78, p = 0.002, df = 1). The H_0_ expectation was derived from the naïve foragers that were added as colonies were hived as swarms at location B (see above). These bees never learned location A and distributed themselves (n = 20) equally between A, C and D (χ^2^ = 0.00, p = 1.00, df = 1; χ^2^ = 0.34, p = 0.56, df = 1; χ^2^ = 0.17, p = 0.68, df = 1 for the 3 replicates, respectively). Thus, this experiment demonstrates that well matured to old foragers can express a spatial memory that was acquired previously, which is distinct in comparison to naïve, inexperienced bees that did not show a spatial orientation preference.

For the laboratory analysis, only foragers from trap boxes A and C were contrasted. Subjects from D were not included due to low sample number (n = 3).

**Figure 4:**
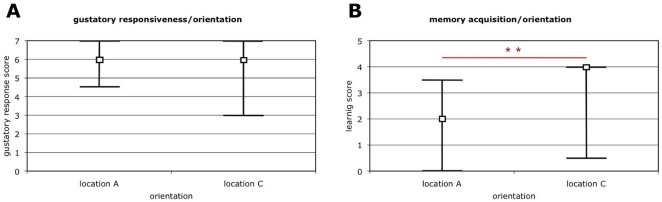
Deficits in olfactory learning predict lack of intact extinction performance. (**A**) No association between sensitivity to sucrose (US) and orientation behavior was found. (**B**) In contrast, reduced olfactory learning ability, a population-level characteristic of old foragers (Fig. 1A,B), is a predictor of orientation behavior in a cohort comprising mixed-age forager groups. Workers that oriented towards a novel location C had the higher median learning ability, outperforming the workers that oriented towards location A, the location bees were trained to extinguish. Graphs indicate medians and interquartile ranges, the asterisk denotes significance (Kruskal-Wallis test).

Figure 4B shows that in well-matured to old foragers, learning performance was a significant predictor of orientation (Kruskal-Wallis H = 16.32, p = 0.006, df = 5), in that workers captured at the previous location A showed lower median learning performance. In addition, a larger proportion of the bees captured at the novel location C learned the CS-US association (successful learners collected from C: 75% vs. A: 57%). These results demonstrate that, by exploiting heterogeneity of learning function in a mixed-age population, we could establish an association between performance values across two different memory tasks: the extinction of a previous abandoned nest location (box A) and the ability to form a novel olfactory memory when the bees are restrained in the laboratory.

Finally, we controlled for spatial orientation of bees and their subsequent memory acquisition in the laboratory was not confounded by gustatory sensitivity. Gustatory sensitivity does not correlate with foraging age [14], [15], but it can be affected by colony environment [35]. This environment was standardized between the trap boxes before the orientation of focus group foragers was tested. Yet, our standardization could not be fully maintained during the last experimental stage because boxes received different numbers of these homing foragers (i.e. nest location A was preferred, Fig. 3.e5). Gustatory sensitivity was tested with an established gustatory response score (GRS) assay [36]. We found no association between GRS and orientation (Kruskal-Wallis H = 6.82, p = 0.44, df = 7, Fig. 4A). This outcome implies that GRS cannot account for the bees' orientation patterns, nor can GRS account for differences in olfactory learning performance between the alternative nest locations.

## Discussion

This study evaluates patterns of associative olfactory learning, memory formation and extinction in old forager bees. To our knowledge, it represents the first individual-level analysis of invertebrate aging that combines inference from composite laboratory data with performance estimates from free-flight. The resulting data reveal a complex performance decline with similarity to aspects of mammalian brain aging.

### Old bees are characterized by poor learning performance and increased performance heterogeneity

We confirmed that old forager bees are characterized by a significant loss of function in memory acquisition when tested in the laboratory. This pattern was established for olfactory learning by Behrends et al. [14] and for tactile learning by Scheiner and Amdam [15]. It is also consistent with studies on other invertebrate species, e.g., the cockroach *Periplaneta americana* [37] and *Caenorhabditis elegans* [38]. However, we extend on these findings by examining the heterogeneity of memory acquisition performance in old bees vs. mature controls. In humans and rodent models, behavioral performance is, likewise, more variable among the old than between individuals that are not yet aged [2], [3]. Such heterogeneity has been attributed to differences in genetic risk factors and to variation in facultative (environmentally inducible) life history traits. However, the emergence of heterogeneity for locomotory behavior and several biomarkers of cellular decline were also observed in isogenic populations of *C. elegans* [39] that were reared in seemingly equal environments. The data, therefore, were explained by a major contribution of stochastic events with effects on patterns of age-related physiological deterioration. In consequence, the progression of aging would be largely unpredictable even when risk factors are known [6], [40]. We believe these ideas will be enriched by data on complex behavioral functions, and that our results exemplify how relevant information can be obtained.

### Are retention and extinction of consolidated memory not affected during honey bee aging?

In contrast to memory acquisition, where old bees performed more poorly than controls, we could not detect age-related differences in LTM retention two days after initial acquisition. Hence, in contrast to a previous study in *Drosophila*, which showed that memory scores shortly after conditioning were not influenced by aging, but LTM was, [13], our data does not lend support for retention of consolidated memory being specifically affected in old honey bees. Yet, apart from true biological differences, these contrasting outcomes from two insect models may also be attributable to specifics of the different experimental paradigms applied, which include the different time lines of retention tests and the use of aversive stimuli in *Drosophila*.

Further, more similar performance of the two age groups after consolidation on day 3 as compared to acquisition day1 may indicate that in honey bees early events of memory formation or the expression of the learned response are stronger affected by aging than late events of memory formation and consolidation. Yet, a direct comparison of events on day 1 and day 2, is problematic, as mortality was considerably higher in the old bees, and the data points for acquisition (Fig. 1C, day 1) and for retention (Fig. 1C, day 3) only partially represent the same individuals. This might bias the results for tests on consolidated memory for this group towards more capable individuals (compare also results section). For the honey bee, however, our findings corroborate a previous assumption made by Scheiner and Amdam [15], who used a slightly different protocol to test tactile memory. Their experiment also did not detect differences between aged bees and controls in LTM retention two days after acquisition. Unlike the present study, however, the design by Scheiner and Amdam could not decouple memory retention from extinction, since memory was retrieved at several intermediate time points by presenting the unrewarded CS.

### In a mixed-age population of mature to old foragers decline of olfactory learning correlates with a decline in spatial extinction ability

To our knowledge, the present study is the first to establish an age related decline of extinction learning in honey bees that move freely in their natural habitat. Our data, thus, suggest similarities with the well-documented extinction decline in freely moving rodents (Morris' water maze) [41]. In contrast, differences for extinction of olfactory memory under restrained conditions were not significant. A decline in behavioral extinction performance in restrained insects, however, was shown before in *Drosophila* [42], [43].

Studies of spatial memory invoke responses that are fundamentally different from those of non-spatial, Pavlovian conditioning [44], involving for example a map-like representation [29], [45] and adult neurogenesis [46], [47]. However, aging may affect multiple systems and functions concomitantly. Our study shows that non-contingent memory functions, i.e. spatial and olfactory learning, can correlate. We found that poor associative learning performance in the laboratory predicted orientation preference towards a location that bees were trained to extinguish (location A). In contrast, individuals that performed better in olfactory memory acquisition had preferentially oriented towards a novel location (C), a behavior that is in compliance with intact extinction performance.

Other influences than memory or memory extinction can bias the orientation of bees to a nest location. Because the release of guidance pheromones and visual cues can affect orientation, we carefully controlled for visual cues and odors on the surface of nest boxes in our experimental setup (see Material and Methods). However, lesser known factors can only be tested and excluded by using an appropriate probe. In our experiment, this probe was represented by the naïve bees that never experienced the hive translocation from A to B. I.e., if orientation preference was influenced by factors other than the experience of different locations then the final distribution of naïve bees would not have been uniform over the alternative locations. Rather, we show that the naïve bees lacked the spatial preference pattern that we observed in the focus group of marked, aged foragers, and thus, we can conclude that orientation was not confounded by uncontrolled factors.

Whereas orientation towards the previous location A strictly excludes intact spatial extinction, the orientation of focus bees towards the novel location C can, in principle, be explained by alternative assumptions. These are intact extinction performance as well as the deletion of the memory of the previous location A. Two lines of evidence, however, support intact extinction for the bees that choose C. First, a previous study also tested the orientation of bees towards a previous nest location [30], and demonstrated that spatial memory of a hive location can last for up to 5 weeks, even without further reinforcement. This persistence of spatial memory is well beyond the time window given for reorientation (Fig. 3.e4) in our study. Second, it is unlikely that functional deterioration, such as “forgetting” should preferentially affect the group (captured at C) that subsequently exhibited the most intact olfactory learning performance in the laboratory. Rather, we show that a measure of functional integrity, as assayed in the laboratory, is significantly reduced in the group whose orientation behavior towards A is in accordance with extinction learning loss. Lastly, reduced performance as measured in the laboratory, also, does not lend support for orientation preference towards A to be explained by better spatial LTM retention in bees choosing the previous location A. Poorer extinction, rather than better LTM retention is further corroborated by experiment 1, which did not indicate an age related effect or even an increase in olfactory memory retention. However, to rule out alternative factors, such as better LTM or better acquisition of the reestablished hive at position A, future studies may directly compare acquisition and memory performance of spatial information during free flight with olfactory learning and LTM retention.

On a final note, our findings do not demonstrate or imply that the performance of honey bees, or their brain functions, decline universally during aging. In fact, a recent study suggests that the regional and molecular units of higher order brain functions can age at different rates in honey bees [33]. However, in the present study we report aging patterns that share similarities with functional aging in other species, including mammalian brain aging. Our study exemplifies that aging can affect different brain functions within the same invertebrate animal. Second, we show that groups of old invertebrates are characterized by increased performance heterogeneity, with some old individuals even performing excellently. Compared to chronological measures (age), screening with established biomarkers of behavioral aging, may therefore improve the resolution also of studies that are concerned with identifying the molecular mechanisms involved in brain aging. This will contribute to better understand aspects of invertebrate aging that have received less attention so far, in comparison to invertebrate life span studies.

## Materials and Methods

### Experiment 1: In-lab testing of olfactory learning, long-term memory retention, and extinction learning of aged bees and controls

#### Subjects and general procedures

Specimens from two *Apis mellifera carnica* colonies were used in this experiment.

Foraging duration is a major determinant of mortality and functional decline in honey bees [36] and, accordingly, the different forager groups were acquired as follows.

Mature control (foraging duration 5–10 days): Within one day after emergence, bees were collected from combs, which were kept in an incubator at 34°C. Bees were then marked with a paint dot on the dorsal thorax. The color code specified the day of emergence and the source colony. Subsequently individuals were released into the hives they were originating from. Hives were continuously observed for marked worker bees to start foraging behavior. After about two weeks, first cohorts of marked bees changed from nest to outside activities, and were caught when returning from their first foraging flights. Individuals were briefly anaesthetized with CO_2_ (<15 sec) and re-marked with a second color tag specifying the day of foraging onset. Care was taken to only shortly expose animals to CO_2_, i.e. within a time window that was previously demonstrated not to induce long-lasting side effects [48]. Animals were captured for behavioral scoring between days 5 to 10 after foraging onset.

Old foragers after extended periods of foraging (foraging duration >15 days): Foragers of random foraging age were caught at the hive entrance. The anaesthetizing and marking procedure was similar to bees marked upon foraging onset (young group). Bees were then allowed to forage for at least 15 more days after marking.

On the evening before the first test sessions (8–9 p.m.) marked bees of both age groups were collected from the hives and introduced into wooden boxes. Bees had access to sucrose *ad libitum* (30%) for 3 hours. Conditioning experiments were started the following day after bees were starved for at least 6 hours. Animals were harnessed in polyacryl holders and, as with all other laboratory tests, were randomized, so that observers were blind to treatment identity. Subsequently, individuals were collectively tested for learning acquisition.

For restrained individuals, several studies reported considerable mortality rates during long-lasting memory tests, thus leading to an unequal distribution of sample sizes across different groups [21], in particular affecting older bees [49] (see also results section). To ensure a high survival during the two days between learning acquisition and memory retrieval tests, we tagged animals individually with numbered plates and re-released them into wooden boxes. All animals that were accidentally harmed by tagging and transfer procedures were discarded. During the two days, bees had access to sucrose (30%) *ad libitum*. Mortality in both forager groups was comparably low, not exceeding 25%, but differed between the age groups.

Upon memory retrieval at day 3, bees were starved again for at least 6 hours before mounting and testing memory retention and extinction.

#### Olfactory learning

Using the classical conditioning of the PER, bees were trained to associate an odor (CS, carnation oil) with a sucrose reward (US, 30% sucrose in H_2_O). We applied a protocol that is shown to specifically induce all phases of LTM formation [26] with inter-trial intervals of 15 min. Prior to learning trials, all individuals were tested for responsiveness by touching the antennae with a droplet of 20% sucrose. Animals that failed to extend their proboscis during US application (n = 18 of n_total_ = 332) were discarded to ensure that poor responsiveness to the US did not confound the measurements of acquisition learning scores.

For conditioning, bees were placed in front of an exhaust fan (10 cm diameter). The CS was delivered through a 10 ml syringe and was presented for 5 seconds. Three seconds after odor onset the US was applied by touching the antennae with the moist tip of an Eppendorf pipette, containing the sucrose US that bees were immediately fed with upon extension of the proboscis (1 µl of 30% sucrose in H_2_O). Bees that stopped responding to the US during learning trials were discarded. A bee was scored positive when extending the proboscis (PER+) within the time window before US application. The learning score (LS), a quantitative measure of acquisition performance, was expressed as the number of CS presentations to which subjects responded, even before the US reward is applied [14]. Because bees were subjected to a total of six CS-US pairings, the LS spans from 5 (bees that learn well) to 0 (no expression of the learned response). Bees that responded spontaneously to the CS alone in the first learning trial were not considered (n = 20 of n_total_ = 332) for calculation of the LS (data for Fig. 1 A,B). For comparison of acquisition and retention behavior, however (data for Fig. 1C,D), where spontaneous responders on day 1 were also tested on day 3, we calculated the median LS for individuals with LS [0–5] as well as for individuals with LS [0–6]. The latter included bees with spontaneous response in learning trial 1. Prior to the first and following the last CS-US pairing, bees were stimulated with cineole, an unrewarded odor (CS-) to distinguish between non odor-specific learning and acquisition of the specific CS-US pairing. In both groups, the percentage of bees responding after training to the unrewarded CS- was less than 10%.

Tests for memory retention and extinction learning were performed on a subset of bees originating from 2 replicate hives. Individuals were subjected to six CS only trials using carnation oil two days after conditioning. The first CS presentation served to test LTM. The total of six CS only presentations served to extinguish the learned response of the CS-US pairing that took place on day 1. The PER was monitored similarly to what was described for memory acquisition.

### Experiment 2: “Homing” performance of aged bees during free-flight correlates with olfactory learning performance in the laboratory

#### Subjects and general procedures

Due to considerable manipulations, we utilized three colonies of Buckfast bees (a hybrid of *Apis mellifera mellifera* and *A. m. ligustica*), a breed of honey bee known for comparably low swarming probability.

The disruptive nature of swarm experiments and concomitant loss of animals renders marking and retrieval procedures described under experiment 1 unfeasible. However, behavioral performance decline was repeatedly shown to correlate with foraging duration (this study, compare also [14], [15]). Thus, similar to other animal systems declined performance values are strongly overrepresented in old cohorts and can be used to screen for individuals with behavioral profiles typical for an old phenotype. Therefore, the marking scheme in this experimental unit was designed to provide a fully mature population of foragers, in which age-related behavioral performance was heterogeneous. At an apiary several kilometers away from the training arenas (see below), foragers were randomly marked in two rounds, on day 1–3 and again on day 7 (Fig. 2C and Fig. 3.e1). When tested in the laboratory at the very end of the experimental protocol, these marked bees had been foraging for a minimum of 15 and 11 days, respectively.

On day 8, the colonies with marked foragers were moved to separate training arenas, each with four hives arranged in a square formation, ten meters apart from one another. The experimental colony was placed at hive location A (Fig. 2B and Fig. 3.e2). The three remaining locations, B, C, and D, were occupied by closed “dummy” hives. To exclude different visual recognition clues at the hive's entrance areas [50], these “dummy” hive boxes were of similar appearance to the bees' native hive. The foraging bees were allowed to learn the larger scale spatial setting of their hive location and empty “dummy” hive boxes for 4 days.

Afterwards, the entire experimental colony was transferred to a hive box location B, which was in the position of a previous “dummy” hive (Fig. 3.e4). To achieve this transfer, we first created an artificial swarm using Robinson and Dyer's method [30]. First, the queen was removed from the hive and placed in a plastic cage. The cage was attached to a mesh screen hanging on a metal cross. A small bottle, containing sugar water, was taped to the cross upside down allowing the sugar water to drip on the queen cage and the cross. Then, the hive was disassembled while applying a vigorous amount of smoke. The smoke forced the foragers to fly up, where they were attracted to the cross with the caged queen and sugar water (Fig. 2A and Fig. 3.e3). Bees that did not fly up were brushed into a plastic tub. For each replicate experiment a set of naïve bees (1000 total) was collected from a distant apiary and anesthetized using CO_2_. Once chilled, they were tagged with a paint mark on their abdomens and introduced into the swarm. The swarm sat on the iron cross over night, while the remaining bees (i.e. nest bees that did not fly to the cross), the brood and food combs were stored on top of a host colony.

The next morning, these bees, the original brood and the food combs were placed into a new box at location B. Bees from the cross (foragers and naïve) were brushed into this hive box as well (Fig. 3.e4). At location B, the colony was allowed to orient and forage for 4–6 days. During this time the now empty, original hive body remained closed at location A.

Following this sequence of colony translocations, the arena was prepared for testing the individual orientation preference of marked foragers towards locations A, C and D. The “dummy” boxes (C, D) and hive box at the previous location A were replaced with identical, new hives, similarly filled with an unrelated queen, young workers (<48 h old), brood, and food combs. Biasing effects of kin and hive recognition cues (queen: [51]; nest mates: [52]; nesting material: [53]) were thereby eliminated. Furthermore, workers may be guided to a nest location by other bees that, upon inspecting the nest, and return to the hive entrance to secrete pheromone [54]. To better ensure that our assay tests individual orientation preference, and not a mass guidance phenomenon, we reduced the probability of pheromone release by using a trap mechanism in all hive boxes. After entering the hive, the bees could not return to the entrance to guide others.

During testing, the queen was removed from location B and the entire worker population of the colony was dumped onto the ground (Fig. 3.e5). Bees were both shaken and brushed off each comb, which forced them to choose a new location to migrate to. The day after, marked bees were sampled from each of the locations A, C and D (Fig. 3.e6), and individuals were tested for olfactory memory acquisition in the laboratory.

#### Gustatory responsiveness and olfactory associative conditioning

Gustatory response scores (GRS) were tested to control for effects of colony environments in the hive boxes bees were collected from. Following the procedures described in [14], each individual was tested for GRS by presenting each bee on the antennae with a drop of sucrose solution and subsequently monitoring PER. The sucrose concentrations were presented to each individual in ascending order: 1, 0.3, 1, 3, 10 and 30%. Olfactory associative conditioning was then performed (for specific methods, refer to experiment 1).

#### Statistical analysis

The data on individual learning performance and gustatory responsiveness were not normally distributed and, therefore, non-parametric tests were used to compare median scores. The differences in the number of bees that displayed specific behavior (i.e., PER+ vs. PER−, or orientation to defined nest locations) were tested by Chi-square and McNemar χ^2^analysis of frequencies. Heterogeneity differences were measured using F-tests on the variances. The F-test is a parametric test without a non-parametric alternative. Analyses were conducted using Statistica 6.0 (StatSoft).
